# Flagellin Delivery by *Pseudomonas aeruginosa* Rhamnolipids Induces the Antimicrobial Protein Psoriasin in Human Skin

**DOI:** 10.1371/journal.pone.0016433

**Published:** 2011-01-25

**Authors:** Ulf Meyer-Hoffert, Alexandra Zimmermann, Manfred Czapp, Joachim Bartels, Yulia Koblyakova, Regine Gläser, Jens-Michael Schröder, Ulrich Gerstel

**Affiliations:** Department of Dermatology, University Hospital Schleswig-Holstein, Kiel, Germany; Fundação Oswaldo Cruz, Brazil

## Abstract

The opportunistic pathogen *Pseudomonas aeruginosa* can cause severe infections in patients suffering from disruption or disorder of the skin barrier as in burns, chronic wounds, and after surgery. On healthy skin *P. aeruginosa* causes rarely infections. To gain insight into the interaction of the ubiquitous bacterium *P. aeruginosa* and healthy human skin, the induction of the antimicrobial protein psoriasin by *P. aeruginosa* grown on an *ex vivo* skin model was analyzed. We show that presence of the *P. aeruginosa* derived biosurfactant rhamnolipid was indispensable for flagellin-induced psoriasin expression in human skin, contrary to *in vitro* conditions. The importance of the bacterial virulence factor flagellin as the major inducing factor of psoriasin expression in skin was demonstrated by use of a flagellin-deficient mutant. Rhamnolipid mediated shuttle across the outer skin barrier was not restricted to flagellin since rhamnolipids enable psoriasin expression by the cytokines IL-17 and IL-22 after topical application on human skin. Rhamnolipid production was detected for several clinical strains and the formation of vesicles was observed under skin physiological conditions. In conclusion we demonstrate herein that rhamnolipids enable the induction of the antimicrobial protein psoriasin by flagellin in human skin without direct contact of bacteria and responding cells. Hereby, human skin might control the microflora to prevent colonization of unwanted microbes in the earliest steps before potential pathogens can develop strategies to subvert the immune response.

## Introduction

Our skin is constantly challenged by microbes but is rarely infected. On intact skin bacterial growth is controlled by the combined action of complementary systems. At the outermost border, the *stratum corneum* layer, a lipid-rich matrix with embedded corneocytes, constitutes a physical barrier with a protective surface pH, nutrient limitation, and physical removal from body surfaces by desquamation. Bacteriostatic and bactericidal compounds such as antimicrobial proteins (AMPs) provided by a keratinocyte derived “biochemical barrier” support this protective function at the skin surface. Once this barrier is disturbed, bacteria or bacterial factors have access to living epidermal keratinocytes and can provoke a host defense program. Irrespectively of manipulating strategies of various bacteria to escape from immune response, finally the sum of effectiveness of the host responses determines if a bacteria is commensal or pathogen.

AMPs are of major importance in maintaining an intact skin barrier against potentially invading microorganisms and in balancing the natural commensal flora on skin. AMPs of the skin, such as the human β-defensins (hBD)-1-3, RNase7, the cathelicidin LL-37, and psoriasin (S100A7c) comprise a heterogeneous group of molecules that use different mechanisms to kill microorganism [1]–[3]. Studies in mouse models with deficiencies in some AMP genes underline the importance of these molecules in protecting the host epithelia against bacterial infections [4]–[6]. One of the dominating AMP of healthy human skin is the S100 protein psoriasin [7] that shows antimicrobial activity *in vitro* preferentially against *E. coli* and other Gram negative bacteria [8]. The physiological role of psoriasin protecting the skin against *E. coli* colonization and infection was underlined by *in vivo* experiments using neutralizing antibodies. Subsequent studies in cultured human keratinocytes identified the Toll-like receptor (TLR)-5 ligand flagellin as “pathogen-associated molecular pattern” (PAMP) of *E. coli*, responsible for the expression of psoriasin mRNA and protein [9].

Although not belonging to the typical resident skin microflora, *P. aeruginosa,* a soil and water-born Gram-negative bacterium is commonly found at different locations of healthy individuals [10]. Here it is usually not infecting healthy skin. In patients suffering from burns, chronic wounds, surgery, and injuries associated with a disruption or disorder of the barrier function of the skin, however, *P. aeruginosa* is able to cause severe infections. The flexible, non-stringent metabolic requirements enable *P. aeruginosa* to occupy a diversity of ecological niches, and in combination with the high resistance to a large number of disinfectants and antibiotics, *P. aeruginosa* emerged to be one of the main Gram-negative bacterium associated with nosocomial infections [11].

To sustain unfavorable conditions, survival of *P. aeruginosa* at surfaces is often interconnected with a switch from the motile planktonic way of life to a structured community of bacterial cells enclosed in a self-produced polymeric matrix. Rhamnolipids, biosurfactants produced by *P. aeruginosa*, play an important role in the initial phase of this biofilm formation [12] by maintaining channels in the biofilm as well as in the dispersal of cells from the biofilm [13]. Additionally, rhamnolipids display excellent antimicrobial activity against a wide variety of Gram-positive and Gram-negative bacteria as well as fungal species [14], [15] and therefore might support *P. aeruginosa* in competition for new habitats.

Rhamnolipids are composed of mono- or di-rhamnose linked to 3-hydroxy-fatty acids of different length, produced during stationary phase of growth. On the genetic level the biosynthesis of rhamnolipids in *P. aeruginosa* is dependent on the *rhlAB* operon, controlled by quorum sensing [16]. Rhamnolipids can be expressed in a concerted manner with different factors and are considered to be important virulence factors [17]. Due to their amphiphilic character, rhamnolipids particularly enhance the biodegradation of hydrophobic compounds such as lipids by solubilization within micelles, thereby increasing the solubility in aqueous media and finally promoting the uptake by the bacterium [18]. Depending on the intended use, *P. aeruginosa* can produce about 30 different congeners of rhamnolipids to meet the required physico-chemical properties [19]. In addition, rhamnolipids are able to modulate the outer leaflet of *P. aeruginosa* mainly by removing lipopolysaccharide [20], which results in increasing hydrophobicity [21], thereby minimizing the distance to potentially hydrophobic substrates/surfaces, for instance the *stratum corneum*. Recently, we could demonstrate that rhamnolipids can shed the PAMP flagellin, the main constituent of the flagellum [22]. A single polar flagellum provides *P. aeruginosa* motility and allows chemotaxis. Especially in airway epithelia the role of flagellin in the pathogenesis of respiratory tract infections is well investigated [23], [24]. Thereby, activation of the host's innate immune system is generally mediated by interaction of flagellin and the extracellular TLR-5 [25].

Combining the previous findings that *P. aeruginosa* can utilize hydrophobic substances which might be lipids of the skin with the aid of rhamnolipids [18], the rhamnolipid mediated shedding of flagellin [22], and the flagellin mediated induction of the antimicrobial peptide psoriasin [9], we aimed to analyze the effect of flagellin-induced psoriasin expression in an *ex vivo* skin model and the contribution of rhamnolipids in this process.

## Results

### *P. aeruginosa* flagellin induces psoriasin release in primary keratinocytes

**Figure 1:**
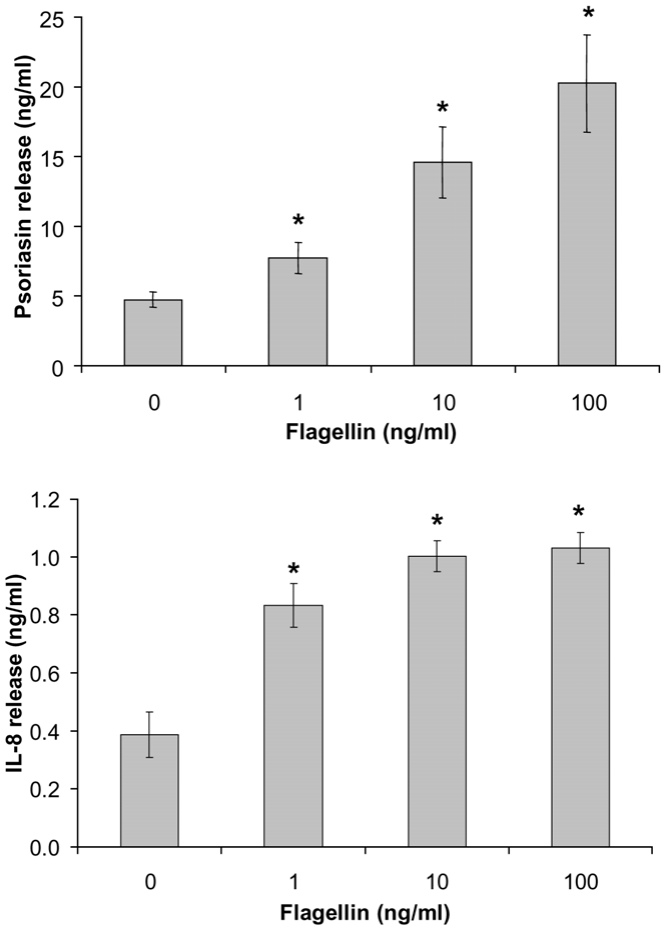
Dose-dependent effect of *P. aeruginosa* flagellin on psoriasin and IL-8 expression. Keratinocytes were stimulated with the indicated concentrations of flagellin for 24 h. Subsequently, psoriasin and IL-8 concentrations of the supernatants were measured by ELISA. Data are means ± SD (n = 3). Stimulation with flagellin increase psoriasin and IL-8 release significantly. * *P<0.05* compared to unstimulated cells (unpaired student's *t*-test).

In order to demonstrate that *P. aeruginosa* derived flagellin is capable of inducing psoriasin and IL-8 in human keratinocytes as described for *E. coli* derived flagellin [9] cultured keratinocytes were stimulated with HPLC-purified flagellin. ELISA-analyses of stimulated keratinocyte supernatants revealed a dose-dependent psoriasin- and IL-8-release that was significantly induced by flagellin at concentrations ≥1 ng/ml (Fig. 1).

### Flagellin-deficient *P. aeruginosa* does not induce psoriasin in human skin *ex vivo*

**Figure 2:**
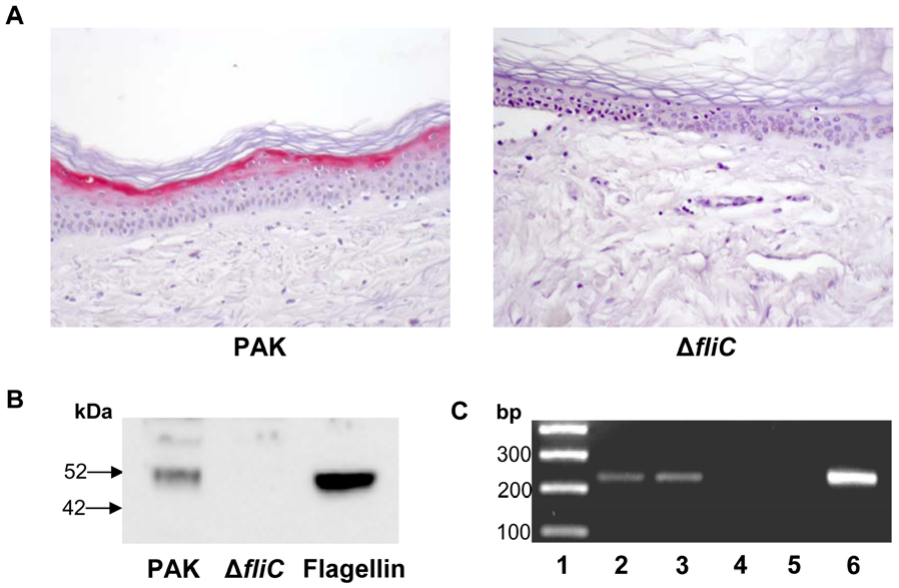
Flagellin is delivered to apical epithelial cells across the *stratum corneum* and induces psoriasin expression. A: Immunohistochemical analyses of psoriasin expression (Vector red) in full-thickness *ex vivo* skin explants. One representative experiment is shown (n = 3–6). Biopsies were incubated with *P. aeruginosa* wildtype (PAK) and its flagellin deletion mutant (Δ*fliC*) in transwells for 24 h. B: Flagellin Western Blot analysis of the correspondent culture supernatants. C: Rhamnolipid expression of *P. aeruginosa* grown directly on *ex vivo* skin was assessed by *rhlA* mRNA expression. A representative gel out of three independent experiments is shown with a 100 bp ladder (lane1), reverse transcription PCR products of PAK (lane 2), Δ*fliC* (lane 3), and untreated skin explants (lane 4), a PCR control for DNA contamination (lane 5) and the PCR product using PAK genomic DNA (lane 6).

Beside flagellin, *P. aeruginosa* might produce other virulence factors during adaptation to certain environmental cues, leading to the induction of psoriasin. To further confirm the role of flagellin as the principal inducer of psoriasin *in vivo*, wildtype flagellated *P. aeruginosa* (PAK) and its flagellin-deficient mutant strain (Δ*fliC*) were cultivated on *ex vivo* skin explants. Both strains were separated from explants by transwells to omit the direct contact between pathogen and skin. Both strains reached similar optical densities at the end of incubation (data not shown). Immunohistochemical analyses revealed an evenly distributed, strong increase of psoriasin expression in the suprabasal epidermal layers of *ex vivo* skin biopsies co-cultivated with the *P. aeruginosa* wildtype strain (Fig. 2a). In contrast, the flagellin mutant Δ*fliC* did not induce psoriasin expression in the skin (Fig. 2a). Similar immunohistochemical analyses were obtained when skin biopsies were directly treated with *P. aeruginosa* or its flagellin-deficient mutant (data not shown). To verify the presence of flagellin under these conditions, bacterial-free culture supernatants were analyzed. As shown in Fig. 2b, Western blot analyses with an anti-FliC antibody revealed the presence of flagellin only in supernatants obtained from the wildtype PAK strain. Based on our hypothesis that rhamnolipids might contribute to the flagellin-induced psoriasin expression in skin, biopsies directly treated with *P. aeruginosa* wildtype or Δ*fliC* mutant were analyzed for the presence of the *P. aeruginosa* derived rhamnolipids by qualitative mRNA expression of the *rhlA* gene, a key gene in rhamnolipid synthesis. As shown in Fig. 2c *rhlA* mRNA could be detected for PAK as well as for Δ*fliC*.

### Rhamnolipids are needed for a flagellin-induced psoriasin expression in human skin *ex vivo*

**Figure 3:**
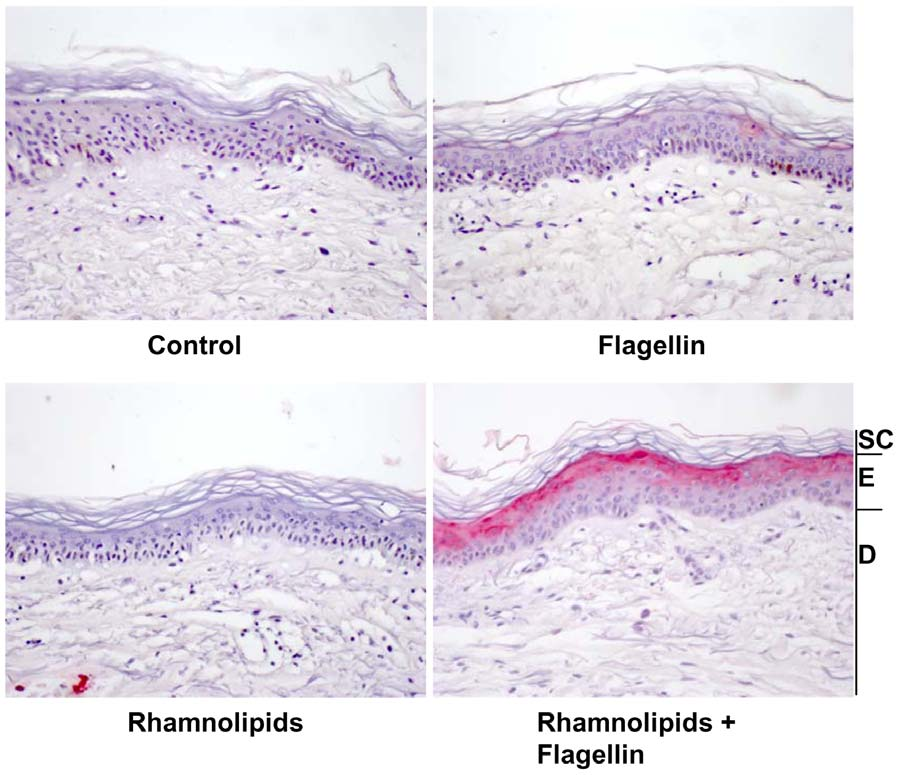
Rhamnolipids enable flagellin-induced psoriasin expression in human skin . Immunohistochemical analyses of psoriasin expression (Vector red) in full-thickness *ex vivo* skin biopsies. Biopsies were incubated with purified *P. aeruginosa* flagellin (700 ng/ml) in the absence and presence of rhamnolipids (37.5 µg/ml) in DMEM for 48 h. One representative experiment is shown (n = 3–6). SC: *stratum corneum*; E: living *epidermis*; D: *dermis*.

In contrast to cultivated keratinocytes, the skin possesses a first, physical barrier with the lipid-rich *stratum corneum* preventing the subjacent cells from damage. *Ex vivo* treatment of skin explants with excess of purified *P. aeruginosa* flagellin (700 ng/ml) and subsequent immunohistochemical analyses showed no expression of psoriasin in the epidermis (Fig. 3).

Considering our observation that *P. aeruginosa* produces rhamnolipids during growth on the skin's surface, the effect of rhamnolipids in combination with flagellin was examined in the *ex vivo* model. Immunohistochemical analyses of psoriasin expression in full thickness skin biopsies revealed a strong increase of psoriasin immunoreactivity in the upper epidermis only when flagellin was applied together with rhamnolipids (Fig. 3). This staining pattern was comparable to that found for skin biopsies co-incubated with PAK wildtype strain (Fig. 2a). Application of rhamnolipids alone did not induce psoriasin expression (Fig. 3).

### Production of rhamnolipids by clinical *P. aeruginosa* isolates

**Figure 4:**
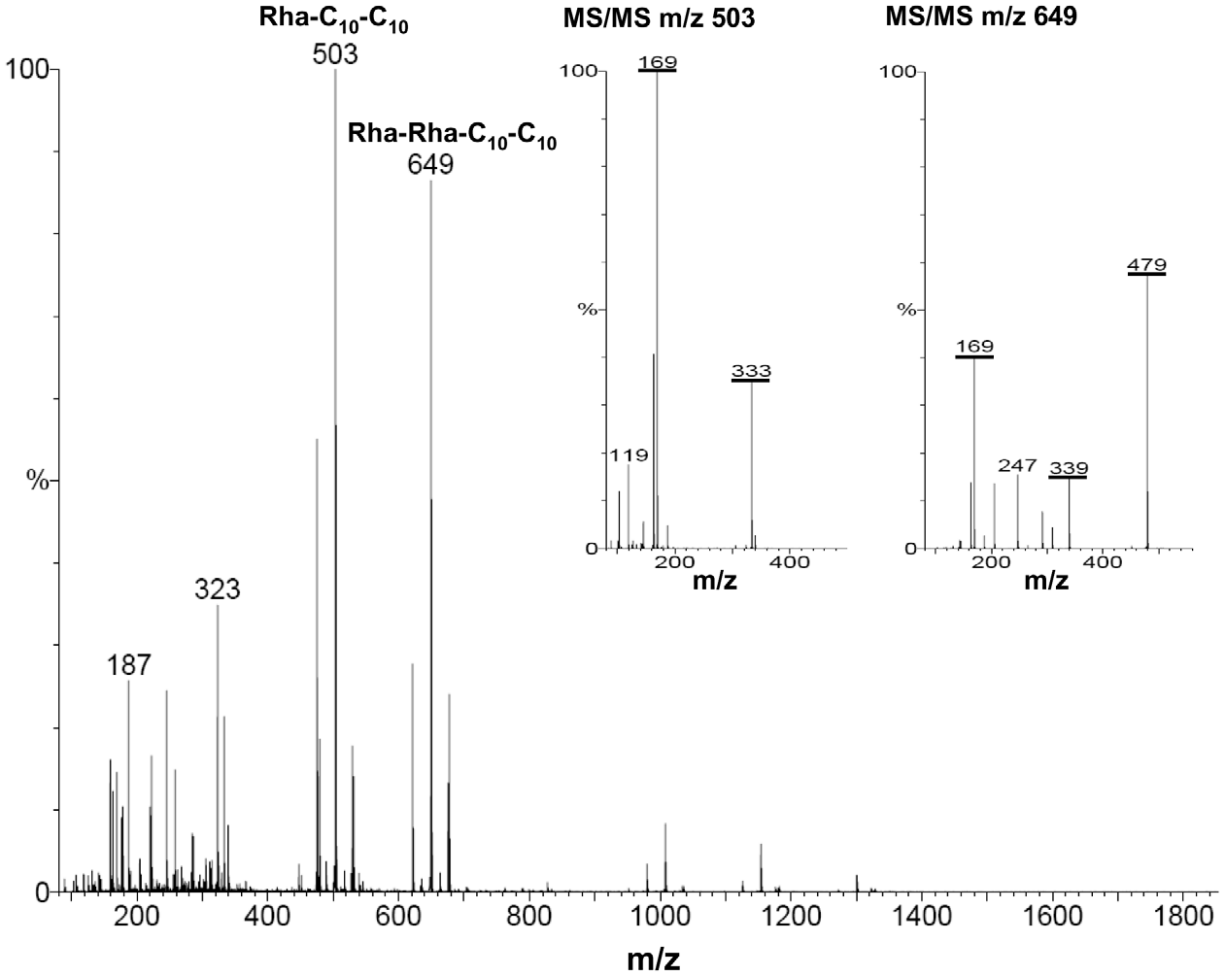
Electrospray-ionization-mass-spectrum of rhamnolipids isolated from the clinical isolate PA264. Analyses of the rhamnolipid preparation were performed in the negative ionization mode. The ion at m/z 503 corresponds to the mono-rhamnolipid Rha-C10-C10 as revealed by MS/MS analyses of m/z 503 displayed in the inset. The ion at m/z 649 corresponds to the dirhamnolipid Rha-Rha-C10-C10 as revealed by MS/MS analyses of m/z 649 displayed in the inset. The corresponding and characteristic ions formed by collision-induced fragmentation are underlined.

To survey whether the flagellin release by rhamnolipids is restricted to laboratory strains, the flagellin and rhamnolipid expression of several clinical *P. aeruginosa* isolates from different sources were analyzed. All of the tested clinical *P. aeruginosa* strains were capable of swimming, indicating an intact flagellum. According to the biochemical approach, tested *P. aeruginosa* strains produced rhamnolipids at concentrations above the critical micelle concentration (CMC) of ∼20 µg/ml, determined for Rha-C_10_-C_10_, Rha-Rha-C_10_-C_10_ [26]. The obtained values vary between 20 µg/ml and 795 µg/ml (Table 1). Since the CMC is dependent on composition of different rhamnolipids [19], electrospray-ionization-mass-spectrometry (ESI-MS) in the negative ionization modus was performed to identify rhamnolipid composition. Figure 4 shows a typical ESI-MS spectrum of the rhamnolipid preparations of the tested clinical *P. aeruginosa* isolates. Prominent ion signals were detected at m/z 503 and 649 and fit well with the MH^-^ of the rhamnolipids Rha-C_10_-C_10_ (calculated mass: 504 Da) and Rha-Rha-C_10_-C_10_ (calculated mass: 650 Da). The obtained fragmentation ions of subsequent tandem mass spectrometry analyses confirmed the presence of two major rhamnolipids (Fig. 4).

**Table 1:** Rhamnolipid production of different motile *P. aeruginosa* strains and clinical isolates.

*P. aeruginosa* isolate (source)	Rhamnolipid concentration (µg/ml)
PAO1	336±78
PAK	38±6
PAKΔ*fliC*	20±1
PA-O (skin)	56±17
PA227 (blood)	63±10
PA264 (skin)	538±76
PA391 (CF-lung)	795±1
PA1450 (skin)	702±25

Rhamnolipids were isolated from 24 h cultures of different *P.aeruginosa* isolates by chloroform extraction and quantified by the Methylene-blue method. The rhamnolipid concentration is given as the mean ± SD of three independent experiments.

### Effect of acetone and different detergents on the flagellin-induced psoriasin expression

**Figure 5:**
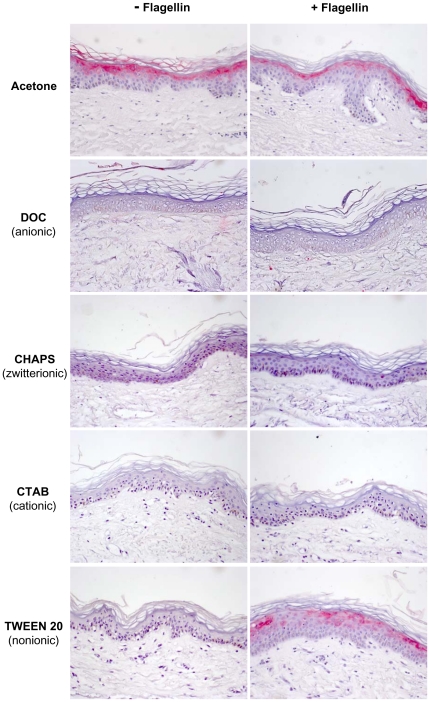
Effects of acetone and different detergents on flagellin-induced psoriasin expression in human skin . For immunohistochemical analyses of psoriasin expression (Vector red) in full-thickness *ex vivo* skin explants, biopsies were incubated with the different surfactants (the anionic detergent DOC, the zwitterionic CHAPS, the cationic CTAB, and the nonionic TWEEN 20) in DMEM for 48 h in presence and absence of *P. aeruginosa* flagellin. Surfactants were added above their CMC. The surfactant net charge is given in brackets. To disorder the physical barrier, *ex vivo* skin was treated with acetone prior incubation with flagellin. One representative experiment out of three is displayed.

To investigate the mechanism of the rhamnolipid mediated psoriasin response to flagellin in the *ex vivo* model, the effect on the epidermal skin barrier was analyzed. As it is known that psoriasin secretion is enhanced after experimental skin barrier disruption [27] the *ex vivo* skin barrier was disturbed by acetone treatment. As shown in Figure 5, acetone treatment itself was sufficient to elicit psoriasin expression in the used model. Stimulation of acetone-treated skin with flagellin resulted in a similar psoriasin expression in the immunohistochemical analyses (Fig. 5).

Rhamnolipids are anionic amphiphilic molecules with detergent properties. Therefore chemically synthesized detergents were analyzed to function as substitute of rhamnolipids in the flagellin delivery across the upper skin layers. The anionic detergent deoxycholate (DOC), the zwitterionic 3-[(3-Cholamidopropyl)dimethylammonio]-1-propanesulfonate hydrate (CHAPS), the cationic hexadecyltrimethyl ammonium bromide (CTAB), and the nonionic TWEEN 20 were applied above their CMC on *ex vivo* skin explants in the absence or presence of flagellin. Subsequently, immunohistochemical analyses of psoriasin expression were performed. As shown in Figure 5, all detergents by their own had no effect on psoriasin expression (left panel). Co-stimulation of flagellin together with DOC, CHAPS or CTAB showed no visible psoriasin expression as revealed by immunohistochemistry (Fig. 5). Solely, analyses of skin that has been stimulated with TWEEN 20 along with flagellin resulted in a visible increase of psoriasin expression, similar to that obtained for rhamnolipids and flagellin (compare Fig. 5 and 3).

### Rhamnolipid mediated induction of psoriasin in human *ex vivo* skin is not restricted to flagellin

**Figure 6:**
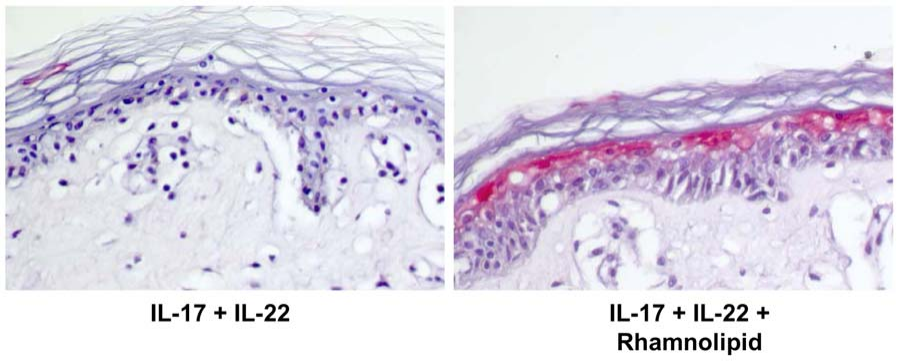
IL-17/IL-22 induced psoriasin expression in *ex vivo* skin is mediated by rhamnolipids . For immunohistochemical analyses of psoriasin expression (Vector red) in full-thickness *ex vivo* skin explants, biopsies were incubated with a mixture of IL-17 (50 ng/ml) and IL-22 (50 ng/ml) in DMEM for 24 h in the absence or presence of *P. aeruginosa* rhamnolipids (37.5 µg/ml). A representative experiment is shown (n = 3).

The previous results assume a rhamnolipid based shuttle system across the *stratum corneum*. To investigate whether a rhamnolipid mediated transport across this barrier is restricted to flagellin or might also be achieved with other proteins, a combination of the cytokines IL-17 and IL-22 was used instead of flagellin. IL-17 and IL-22 are potent inducers of psoriasin and a combination of them has been shown to be more effective in inducing psoriasin secretion in keratinocytes than the two cytokines separately [27]. Immunohistochemical analyses of *ex vivo* skin biopsies, treated with a mixture of IL-17 and IL-22, revealed no detectable psoriasin expression within the epidermis (Fig. 6). In contrast, using this mixture in combination with rhamnolipids, the suprabasal epidermal layer showed distinct psoriasin expression (Fig. 6).

### Rhamnolipids form vesicles and do not enhance flagellin-induced psoriasin expression *in vitro*

**Figure 7:**
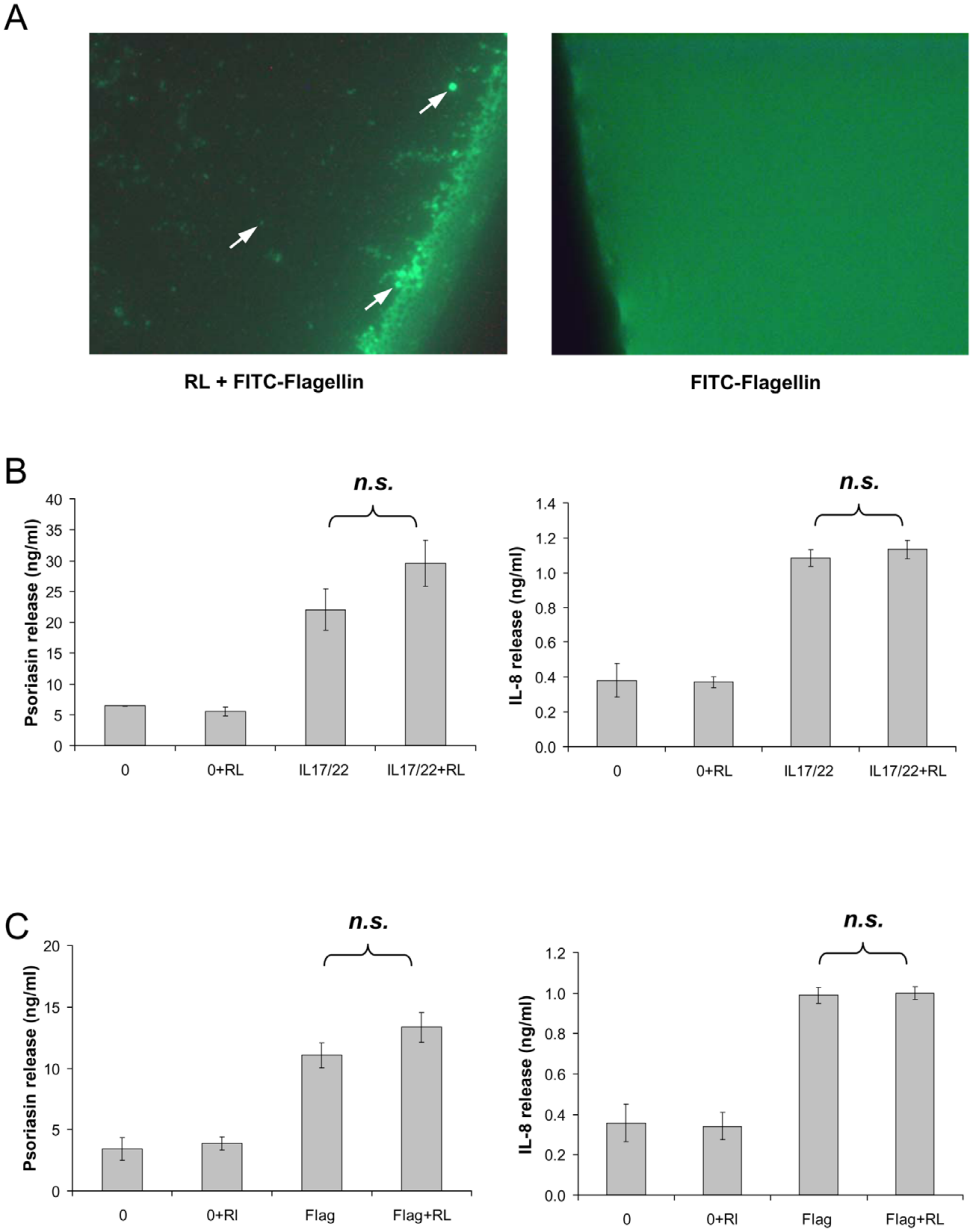
Rhamnolipids form vesicles and do not alter the potency of flagellin and IL17/IL-22 in cultured keratinocytes. A: Representative photomicrograph showing FITC-labeled flagellin embedded in rhamnolipid (RL) vesicle (indicated by arrowhead). On the left hand the control without rhamnolipid (400× magnification) B: Psoriasin- and IL-8-secretion was measured by ELISA in the supernatants of cultured primary keratinocytes after incubation with rhamnolipids (0.05 µg/ml), a mixture of the cytokines IL-17 and IL-22 (both 50 ng/ml), as well as a combination of rhamnolipids and cytokines for 24 h. C: Secreted psoriasin and IL-8 was determined by ELISA in supernatants of primary keratinocytes, stimulated with rhamnolipids (0.05 µg/ml) and/or flagellin (100 ng/ml) for 24 h. The release is given as the mean ± SD (n = 3). The addition of rhamnolipid during stimulation did not alter the potency of the used stimuli (n.s.: not significant) as determined by unpaired student's *t*-test.

Rhamnolipids resemble glycolipids and are able to form micelles or vesicles (liposomes). Therefore vesicle formation and the uptake of FITC (fluorescein isothiocyanate)-labeled flagellin was investigated at pH 5, reflecting the physiological pH of skin. As shown in Figure 7a, rhamnolipids form flagellin embedded vesicles of different size.

Vesicles display potential carrier molecules and may enable a transmembrane transport and a possible translocation of PAMPs into the host cell. Therefore a possible impact of rhamnolipids on flagellin- and IL17/IL-22-induced psoriasin secretion was investigated in cultured keratinocytes. Rhamnolipids were applied at concentrations below CMC, since concentrations above CMC are known to be cytotoxic for cultured keratinocytes [28]. Neither flagellin- nor IL-17/IL-22-induced psoriasin expression was significantly enhanced in the presence of rhamnolipids, as determined by unpaired student's *t*-test (Fig. 7b, c). As shown in Figure 7b and c, rhamnolipids by their own raise neither the psoriasin nor IL-8 release in human keratinocytes at the used concentrations.

## Discussion

*P. aeruginosa* occupies a diversity of ecological niches, last but not least due the versatile and non-stringent metabolic requirements. Thus it is not astonishing that *P. aeruginosa* is quite commonly found on human skin [29], [30]. While intact skin is rarely infected with *P. aeruginosa*, impairment of the skin as burns, chronic wounds, or surgery frequently result in severe infections outlining the protective functions of the epidermal skin barrier.

This prompted us to examine the effects of *P. aeruginosa* and the *P. aeruginosa* derived PAMP flagellin, respectively, when encountering the skin. Both, application of *P. aeruginosa* directly on skin as well as separated from skin by transwells, resulted in an induction of psoriasin in the living epidermis (Fig. 2a). Keeping in mind that flagellin is the principal inducer of psoriasin expression in keratinocytes *in vitro* [9] and that the flagellin deficient mutant did not show any detectable psoriasin expression in *ex vivo* skin (Fig. 2a), highlights the role of flagellin as major PAMP. However, purified flagellin, applied to skin *ex vivo*, did not induce a response of the innate immunity in form of a psoriasin expression in the living epidermis at all (Fig. 3). This raised the question how *P. aeruginosa* derived flagellin is delivered across the dead corneocytes of the *stratum corneum* to responding host cells. The *stratum corneum* confers impermeability towards most solutes and this constitutes a well known problem in transdermal drug delivery that usually can be bypassed by empirically determined compositions of detergents and lipids or liposomes [31]. We speculate that *P. aeruginosa* rhamnolipds as glycolipids act in a similar way. Rhamnolipids display detergent like properties and were secreted by *P. aeruginosa* in the used *ex vivo* skin model (Fig. 2c). Verifying this hypothesis, flagellin applied in the presence of *P. aeruginosa* derived rhamnolipids provoked a psoriasin expression in *ex vivo* skin comparable to that from *P. aeruginosa* wildtype (compare Fig. 3 with Fig. 2a). Rhamnolipids alone had no effect on the psoriasin induction in the used skin model. Our findings support the hypothesis that rhamnolipids act as a shuttle system for flagellin transport across the *stratum corneum*. Although detergent properties of rhamnolipids might mediate the flagellin-transport, this cannot be sufficient as indicated by the failure of most of the tested detergents to facilitate the flagellin-induced psoriasin expression (Fig. 5).In contrast to acetone (Fig. 5) or tape stripped skin barrier disruption [27], the integrity of the *stratum corneum* was obviously not disturbed by rhamnolipids, indicated by the absence of psoriasin expression in rhamnolipid-treated skin. In accordance to this no elevated transepidermal water loss, determined on a standardized area of the forearms of healthy volunteers could be detected (data not shown). This observation implicates a vesicle- or micelle-based delivery system for the rhamnolipid mediated flagellin transport across the *stratum corneum* that does not damage the skin barrier. Supporting this idea, rhamnolipids were shown to form polydisperse vesicles at pH levels around 5 [32], which is the natural pH of the skin surface, and were able to encase FITC-labeled flagellin (Fig. 7a). Ongoing investigation revealed that the rhamnolipid-based shuttle system is not restricted to flagellin. Even host derived factors as the cytokines IL-17 and IL-22 were shuttled by the aid of rhamnolipids assuming that the rhamnolipid-based delivery system could constitute a universal shuttle system throughout the skin barrier. Connecting this universal shuttle system with the excellent antimicrobial activity of rhamnolipids against a wide range of bacteria and fungi [14], [15], it can be assumed that also factors from *P. aeruginosa* competitors or commensals on skin might be delivered across the *stratum corneum* finally resulting in activation of a specific host response. A recent study by Abtin [33] gives reason to speculate that this system is not restricted to *P. aeruginosa*. Here the authors demonstrated that *E.coli* is also able to induce the TLR-5 mediated antimicrobial heterodimer calprotectin on *ex vivo* skin explants.

Our experiments using the flagellin-deficient *P. aeruginosa* mutant highlight the importance of flagellin as a key PAMP. Extracellular recognition of flagellin by the host is generally mediated by TLR-5 and finally lead to the induction of proinflammatory mediators like IL-8 and AMPs [9], while cytosolic flagellin is detected through the NOD-like receptor Ipaf, which leads to the activation of the inflammasom [34]. In addition to the delivery of flagellin into the cytosol via specific virulence factor transport systems (the type III and type IV secretion system), a long distance delivery system for bacterial virulence factors via outer membrane vesicles directly into the host cell cytoplasm was recently proposed by Bomberger and colleagues [35]. Verifiyng that rhamnolipid vesicle may potentially be able to fuse with the cell membrane of the host and release its contents into the cytoplasm, *in vitro* co-stimulations of keratinocytes with rhamnolipid and flagellin were performed but did not significantly enhance the induction of IL-8 and psoriasin (Fig. 7b) under the used experimental conditions. This result suggest that the induction of IL-8 and psoriasin by flagellin seems unlikely to be caused by intracellular recognition.

Researchers generally work with laboratory strains under controlled conditions but this might not reflect the potential of clinical isolates of *P. aeruginosa*. Previous reports have shown that only a minority of tested clinical strains failed to produce rhamnolipids [36], even though rhamnolipids cause the release of PAMPs [20], [22]. The herein investigated clinical isolates did not diminish the production or change the composition of secreted rhamnolipids (Table 1; Fig. 4) although rhamnolipids seem to be an Achilles' heel for *P. aeruginosa* during growth on the healthy skin surface.

**Figure 8:**
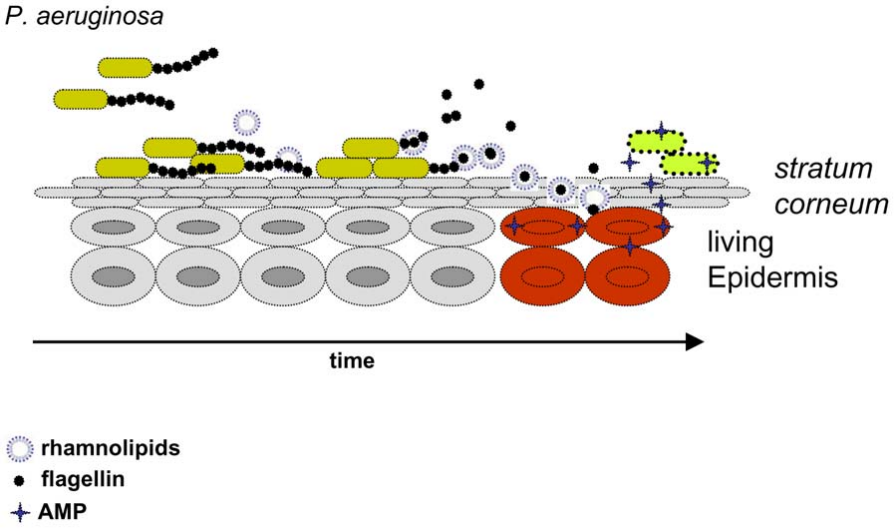
Proposed model for rhamnolipid mediated delivery of flagellin across the *stratum corneum.* Rhamnolipids released from *P. aeruginosa* form micelles or vesicles and shed flagellin. Rhamnolipid embedded flagellin penetrates the human *stratum corneum* and is subsequently released into the living epidermal cell layers. Here flagellin activates keratinocytes (red) and induces the production and release of AMPs, which in turn kill *P. aeruginosa*.

In conclusion, our data suggest that rhamnolipids were produced during initial settlement of *P. aeruginosa* at the skin surface, maybe to eliminate competitors, supporting the biodegradation of the hydrophobic *stratum corneum*, or to establish microcolonies [12]. Consequently, the rhamnolipid secretion caused the release of the main psoriasin-stimulus flagellin [22]. Thereafter flagellin, likely encased by rhamnolipids, was shuttled to the living epidermis via the *stratum corneum*. Consecutively, flagellin causes an induction of an antimicrobial defense response in the uppermost epidermal layers. This early and efficient keratinocyte defense reaction during a most vulnerable phase of the pathogen might explain why *P. aeruginosa* is only a transient constituent of human's healthy skin (Fig. 8).

## Materials and Methods

### Ethics Statement

All experiments were performed according to the Declaration of Helsinki protocols and were approved by the ethics committees of the Medical Faculty of the Christians-Albrechts-University/University Hospital Schleswig-Holstein, Kiel, Germany (AZ 104/06). Skin explants for *ex vivo* experiments derived from patients undergoing plastic surgery after written informed consent.

### Bacterial growth conditions and strains

*P. aeruginosa* strains were maintained as glycerol stocks at −80°C and grown on trypticase soy agar or in trypticase soy broth (TSB) overnight at 37°C under agitation (200 rpm). For rhamnolipid production *P. aeruginosa* was grown overnight at 37°C with agitation (200 rpm) in 1% peptone 1% glucose medium. For swimming assays strains were grown overnight on TSB agar plates at 37°C. Individual colonies were then stabbed onto swimming plates (TSB, 0.3% agar (w/v)) with a sterile toothpick and incubated at 22°C. Swimming motility was determined by measuring the diameter of the turbid zone around the inoculation site. Strain PAK and its Δ*fliC* mutant [37] were obtained from Dr. R. Ramphal (Gainesville, Florida). The laboratory strain PAO1 originate from the Holloway collection. Strains PA227; PA264; PA391,P A1450 and PA-O [38] were obtained from patients at the University Hospital Schleswig-Holstein, Kiel, Germany.

### Cell Culture

Foreskin derived primary keratinocytes were isolated from neonatal foreskin as described [39] and cultured in EpiLife medium (Cascade Biologics) in collagenized 75-cm^2^ flasks (BD Biosciences) in a humidified atmosphere with 5% CO_2_. For stimulation experiments cells were seeded in 12-well tissue culture plates (BD Biosciences) and used at 80–90% confluence. Cells were stimulated with purified flagellin of *P. aeruginosa* PAK, IL-17, and IL-22 (PeproTech) at indicated concentrations and time points.

### RNA isolation, cDNA synthesis, and qualitative *rhlA* PCR

At the end of the stimulation experiment skin samples were minced in liquid nitrogen and RNA was isolated using the NucleoSpin RNA II kit (Macherey&Nagel), according to the supplier's protocol. RNA quality and quantity were determined by photometry. To check for DNA contamination, RNA samples were analyzed by PCR using primers for *rhlA*. Subsequently, 1 µg of total RNA was reverse transcribed into cDNA using random hexamer primers and 50 units Superscript II (Invitrogen) according to the manufacturer's protocol. Qualitative *rhlA* PCR was performed with the *rhlA* forward primer 5′ CGA AAG TCT GTT GGT ATC GG 3′, *rhlA* reverse primer 5′ CGT CCT TGG TGA TCA ACC C 3′ and cDNA corresponding to 20 ng RNA as template in a 25 µl reaction.

### ELISA

The psoriasin ELISA was performed as described previously [8]. IL-8 was detected in keratinocyte culture supernatants using a commercially available IL-8 ELISA (R&D Systems).

### Rhamnolipid purification and quantification

Bacteria grown to an OD_600nm_ of 2-2.5 were pelleted by centrifugation and the supernatant was sterile filtrated and acidified with HCl to pH 3. Rhamnolipids were extracted twice by addition of two volumes ethylacetate. The pooled organic fractions were vacuum-dried and the lyophilizated sample was dissolved in distilled water and analyzed by ESI-MS. Quantification of rhamnolipids in the culture broth were determined by complexation of rhamnolipids and Methylene-blue as described [40].

### Rhamnolipid vesicle

Rhamnolipids were dissolved with FITC-labeled flagellin in chloroform-methanol (2∶1 v/v) The solvent was evaporated (Heidolph rotary evaporator) and sodium phosphate buffer (10 mM, pH 5) was added to reach the final concentration of 37.5 µg/ml rhamnolipids. The content of the flask was stirred vigorously at room temperature. The incorporation of the FITC-labeled flagellin into rhamnolipid vesicle was observed by analyses with a microscope (400× magnification). FITC was excited using a 100W HBO mercury lamp in conjunction with a FITC filter (OLYMPUS).

### Mass spectrometry (MS)

For mass analyses purified rhamnolipid preparations were dissolved in 50% acetonitrile-water and characterized by electrospray-ionization-mass-spectrometry (ESI-MS) in the negative ionization modus. The analyses were performed with a Quadrupol-Time-of-Flight-Hybrid-Mass spectrometer (Q-TOF™II, Waters Micromass, Milford, Massachusetts) equipped with an orthogonal electrospray source (Z-spray). For MS/MS analyses nanoelectrospray ionization [41] was used and were carried out after fitting the Q-Tof™II mass spectrometer with a nano Z-spray source.

### SDS-PAGE and Western Blot

The bacterial culture supernatants were boiled in 4× loading-buffer (4% SDS, 60% Glycerol, 0.3 M Tris, 0.01% Coomassie Brillant Blue G-250). Tricine-SDS-PAGE was then performed in 4% stacking and 10% separation gels as described by Schägger [42].

Flagellin was transferred to nitrocellulose membranes (SIGMA) and blocked with 5% (w/v) dried skim milk in 0.05% TWEEN 20 in PBS. Immunoblotting was performed with polyclonal anti-flagellin kindly provided by Dr. R. Ramphal.

### Purification of flagellin

Flagellin was purified from *Pseudomonas* strain PAK grown overnight in TSB. Bacteria were pelleted by centrifugation and resuspended in phosphate buffered saline (PBS). Flagella were removed by heat treatment at 65°C for 10 min, bacteria were pelleted again by centrifugation and supernatants were supplemented with 0.1 M MgCl_2_ (final). Solutions were allowed to incubate over night at 4°C. Precipitated flagellin was then harvested by centrifugation and dissolved in 20 mM TRIS-Cl pH 8. Flagellin-enriched samples were loaded onto Mini Q PC 3.2/3 anion-exchange column (GE Healthcare, Munich, Germany) on an ETTAN™ HPLC system and eluted with linear gradient of 1 M NaCl. Flagella eluted at 0.2 M NaCl as monitored by A_280nm_. The presence of flagellin was confirmed by a characteristic 49 kDa band in silver-stained polyacrylamide gels or immunoblotting using anti flagellin. FITC labeling of flagellin was performed as described by the manufacturer (SIGMA). Separation of FITC labeled flagellin from unbound FITC was performed by using 30 kDa cutoff ultrafiltration devices (Millipore).

### *Ex vivo* stimulation of human skin

Skin explants for *ex vivo* experiments derived from plastic surgery were kindly provided by C. Preissner (Vital-Aesthetic–Clinic Kiel, Germany). Fresh tissue was transferred into PBS and immediately used for experiments. Subcutaneous fat was removed mechanically and skin explants were washed in PBS. Punch biopsies (6 mm diameter) were taken, placed into the wells of 96 well tissue culture plates (BD Biosciences) filled with 100 µl DMEM medium (Cell Concepts) and finally stimulated with flagellin, IL-17/IL22 or a combination of the stimuli and surfactant (rhamnolipids: JBR515 Jeneil Biosurfactant; DOC; CHAPS; CTAB; TWEEN 20, (all SIGMA) at concentrations of 37.5 µg/ml, 3 mg/ml, 4 mg/ml, 1 mg/ml, and 0.1 mg/ml, respectively), and appropriate controls. For disorder of the *stratum corneum*, skin explants were wiped with acetone-soaked swabs before punch biopsies were taken. The explants were incubated for 48 h at 37°C in a humidified atmosphere with 5% CO_2_. Using living bacteria, bacteria were separated from punch biopsies by transwells (NUNC tissue culture inserts, 0.2 µm pore size) and 10000 CFU of PAK or Δ*fliC* were applied. Untreated skin served as control. Incubation was performed for 24 h at 37°C in a humidified atmosphere with 5% CO_2_. For exposure of PAK and Δ*fliC* directly on skin, explants of 1 cm^2^ were placed on DMEM-drained dressing material in 12 well tissue culture wells, and were exposed to living bacteria for 24 h at 37°C in a humidified atmosphere with 5% CO_2_. After incubation, punch biopsies were taken and fixed in 4% formalin over night and embedded in paraffin.

### Immunohistochemical analyses

To investigate psoriasin protein expression in skin explants, paraffin sections were analyzed by immunohistochemistry as described previously [8]. To test the specificity of the used antibody, positive staining derived from rhamnolipid and flagellin co-stimulated skin explants were incubated with the psoriasin monoclonal antibody preabsorbed with 1 µg of skin derived psoriasin (data not shown). Intensity of psoriasin immunoreactivity in the epidermal layer and the corresponding controls were optically evaluated and scored in arbitrary units (Fig. S1).

## Supporting Information

Figure S1 Scoring of the psoriasin expression in immunhistochemistry analyses was accessed by visual judgment of the processed paraffin sections and scored in arbitrary units. (RL: rhamnolipid)
(TIF)
