# Supplementary figures and images for: Flagellin Delivery by Pseudomonas aeruginosa Rhamnolipids Induces the Antimicrobial Protein Psoriasin in Human Skin
Source: PLoS One. 2011 Jan 25;6(1):e16433. doi: 10.1371/journal.pone.0016433 (PMC3026827; doi:10.1371/journal.pone.0016433)

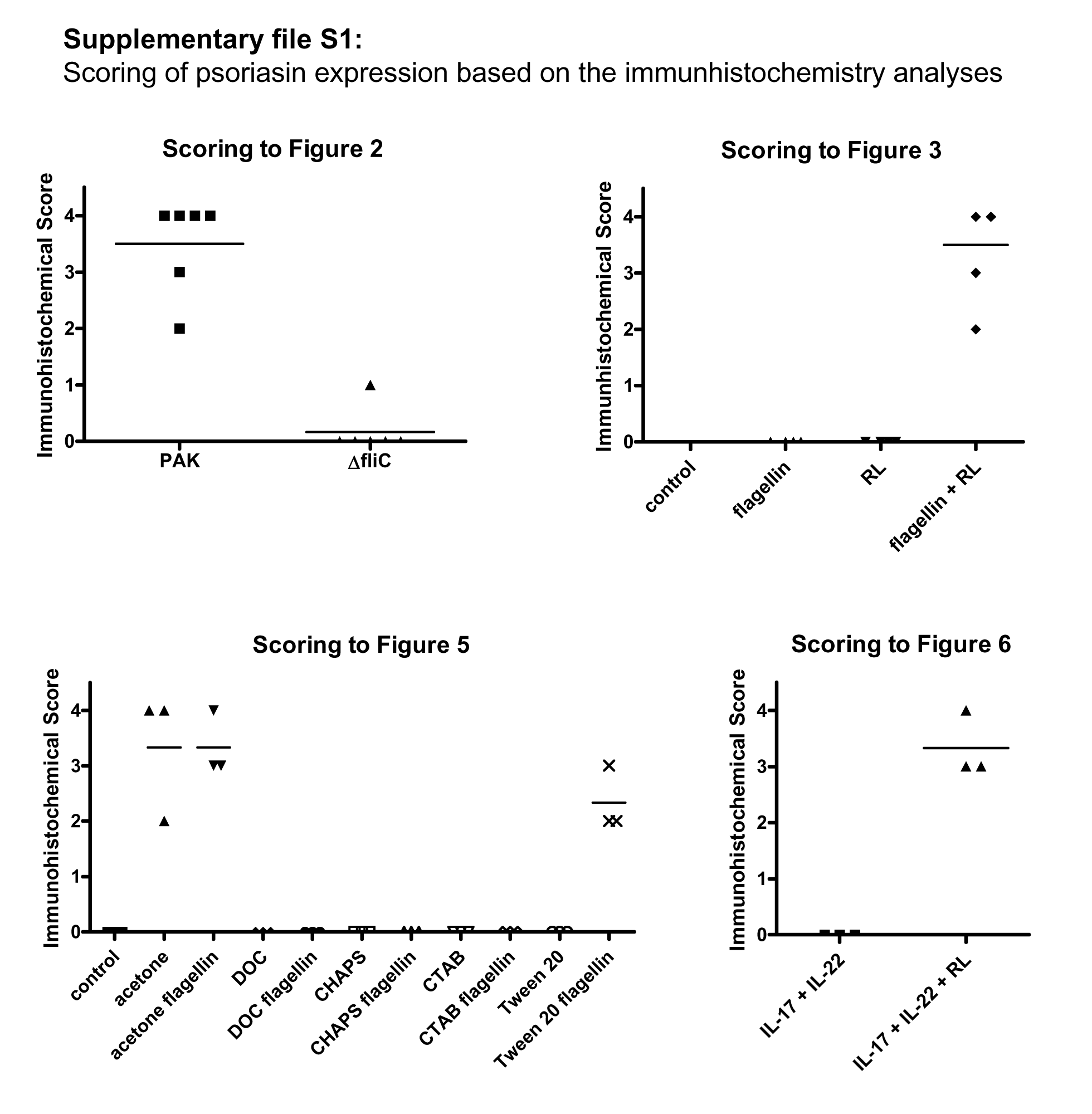

Supplement: Figure S1 — Scoring of the psoriasin expression in immunhistochemistry analyses was accessed by visual judgment of the processed paraffin sections and scored in arbitrary units. (RL: rhamnolipid) (TIF) [file pone.0016433.s001.tif]
